# PCR Improves Diagnostic Yield from Lung Aspiration in Malawian Children with Radiologically Confirmed Pneumonia

**DOI:** 10.1371/journal.pone.0021042

**Published:** 2011-06-14

**Authors:** Enitan D. Carrol, Limangeni A. Mankhambo, Malcolm Guiver, Daniel L. Banda, The IPD Study Group, Brigitte Denis, Winifred Dove, Graham Jeffers, Elizabeth M. Molyneux, Malcolm E. Molyneux, C. Anthony Hart, Stephen M. Graham

**Affiliations:** 1 Department of Women's and Children's Health, The University of Liverpool, Institute of Child Health, Alder Hey Children's NHS Foundation Trust, Liverpool, United Kingdom; 2 Malawi-Liverpool-Wellcome Trust Clinical Research Programme, University of Malawi College of Medicine, Blantyre, Malawi; 3 Department of Paediatrics, University of Malawi College of Medicine, Blantyre, Malawi; 4 Health Protection Agency, Health Protection Agency North West, Manchester Medical Microbiology Partnership, Manchester, United Kingdom; 5 Department of Clinical Infection, Microbiology and Immunology, The University of Liverpool, Duncan Building, Liverpool, United Kingdom; 6 Centre for International Child Health, University of Melbourne Department of Paediatrics and Murdoch Children's Research Institute, Royal Children's Hospital, Melbourne, Australia; Aga Khan University, Pakistan

## Abstract

**Background:**

Accurate data on childhood pneumonia aetiology are essential especially from regions where mortality is high, in order to inform case-management guidelines and the potential of prevention strategies such as bacterial conjugate vaccines. Yield from blood culture is low, but lung aspirate culture provides a higher diagnostic yield. We aimed to determine if diagnostic yield could be increased further by polymerase chain reaction (PCR) detection of bacteria (*Streptococcus pneumoniae* and *Haemophilus influenza*e b) and viruses in lung aspirate fluid.

**Methods:**

A total of 95 children with radiological focal, lobar or segmental consolidation had lung aspirate performed and sent for bacterial culture and for PCR for detection of bacteria, viruses and *Pneumocystis jirovecii*. In children with a pneumococcal aetiology, pneumococcal bacterial loads were calculated in blood and lung aspirate fluid.

**Results:**

Blood culture identified a bacterial pathogen in only 8 patients (8%). With the addition of PCR on lung aspirate samples, causative pathogens (bacterial, viral, pneumocystis) were identified singly or as co-infections in 59 children (62%). The commonest bacterial organism was *S.pneumoniae* (41%), followed by *H. influenzae* b (6%), and the commonest virus identified was adenovirus (16%), followed by human bocavirus (HBoV) (4%), either as single or co-infection.

**Conclusions:**

In a select group of African children, lung aspirate PCR significantly improves diagnostic yield. Our study confirms a major role of *S.pneumoniae* and viruses in the aetiology of childhood pneumonia in Africa.

## Introduction

Pneumonia is the major cause of death in children throughout the developing world especially in children under the age of 5 years [1]. Most deaths attributable to acute respiratory infection are caused by pneumonia and bronchiolitis [2]. Data of the causes of pneumonia are required from regions where deaths due to pneumonia are common, in order to inform case-management guidelines and the potential of prevention strategies such as bacterial conjugate vaccines [3].

An inherent difficulty with pneumonia aetiology studies is the low yield from blood culture. Lung aspiration with culture provides a higher diagnostic yield and is more specific for causative pathogen than blood culture. Lung aspirate is safe and provides reliable diagnostic information [4].

Lung aspirate studies have contributed significantly to our understanding of the bacterial aetiology of pneumonia, and data from these studies provided the important evidence base for the case-management strategy recommended by the World Health Organization (WHO) [2]. However, as is the case for blood culture, the yield from lung aspirate culture is likely to be affected by prior antibiotic usage, especially for the more fastidious organisms, such as *Streptococcus pneumoniae*. PCR of lung aspirate fluid increases the yield further [5], [6] especially in patients who have received antibiotics [7].

The contribution of viruses to the aetiology of pneumonia is uncertain as most studies utilise nasopharnygeal aspirates, and it is therefore difficult to imply causation as virus detection in the nasopharynx may simply reflect colonisation [8]. In South African children, viral pathogens were identified less frequently than bacterial pathogens in HIV-infected children, and HIV-infected children with severe viral lower respiratory tract infections (LRTI) had higher mortality than HIV-uninfected children [9]. In children under 2 years of age with LRTI, most pathogens identified were predominantly RSV, Influenza A+B, Parainfluenzae 1–3 and adenovirus. Polymicrobial disease has been identified as a major cause of treatment failure in children under 5 years of age in South Africa [10].

We performed lung aspiration on a select group of Malawian children as part of a larger descriptive study examining the cytokine profile and genetic determinants of invasive pneumococcal disease. Here, we report the use of PCR for detection of *Streptococcus pneumoniae*, *Haemophilus influenzae* type b (Hib), *Mycoplasma pneumoniae*, *Chlamydia pneumoniae*, *Pneumocystis jirovecii* and nine respiratory viruses: Respiratory Syncytial Virus (RSV), Human metapneumovirus (hMPV), Influenza A and B, Parainfuenza virus 1–4, Coronavirus (HKU-1, NL63, OC43), Rhinovirus, Adenovirus, and Human Bocavirus (HBoV) in lung aspiration specimens, and we compare diagnostic yield for bacteria between PCR applied to lung aspirate and bacteriological culture of blood and lung aspirate fluid. We show that PCR of lung aspirate fluid increases diagnostic yield.

## Results

### Study population characteristics

During the study period, we screened 424 children with a clinical diagnosis of pneumonia by chest radiographs, of which 95 had lobar, focal, or segmental consolidation. We recruited 95 children between 1 April 2004 and 31 October 2006. The median age was 2.6 years, interquartile range (IQR) 1.0–5.7 years. Of the total, 58 were male (61%), 59 (62%) were HIV-infected, and 6 died (6.3%). Characteristics of children with radiologically confirmed pneumonia are shown in Table 1.

**Table 1:** Characteristics of children with radiologically confirmed pneumonia.

Characteristic	Number (%) n = 95
**Age**	
2–5.99 months	9 (9.5%)
6–11.99 months	14 (14.7%)
1–4.99 years	45 (47.4%)
5–9.99 years	20 (21.1%)
10–15 years	7 (7.4%)
Males	58 (61%)
Deaths	6 (6.3%)
Median age- years (IQR)	2.6 (1.0–5.7)
Median duration of symptoms- days (IQR)	4 (3–7)
Median duration of hospital stay- days (IQR)	4 (3–5)
**Prior treatment**	
**Antibiotics**	49 (52%)
Penicillin	20
Cotrimoxazole therapy	15
Of which, on prophylaxis	3
Gentamicin	9
Amoxycillin	4
Erythromycin	3
Chloramphenicol	2
Flucloxacillin	1
Don't know antibiotic	2
**Antimalarials**	13 (14%)
SP	12
Quinine	1
**Clinical features**	
Temperature >38°C	62/95 (65%)
Median temperature - °C (IQR)	38.7 (37.7–39.2)
Increased work of breathing	66/92 (72%)
Grunting	45/95 (47%)
Crackles	72/93 (77%)
Hypoxia (O_2_ saturation<90%)	17/90 (19%)
Median O_2_ saturation % (IQR)	94 (90–96)
Oxygen therapy	28/91 (31%)
HIV-infected	59 (62%)
Wasting (wt-for-ht Z score<−3)	17/76 (9.2%)
Stunting (ht-for-age Z score<−3)	20/91 (22%)

Of the 89 cases in whom immunisation was completely recorded, 82 (92%) were reported to have completed 3 doses of primary immunisation, including Hib. Cases presented throughout the year, but the peak of pneumonia cases occurred between August and November, the hot dry season.

### Pathogens identified by culture or PCR

A pathogen (bacterial, viral, pneumocystis) was identified in 59 children (62%), either singly or as co-infection. Only 10 samples in 9 patients (9%) were positive by culture; 8 by blood culture and 2 by lung aspirate culture. The aetiological agents are listed in Table 2. The commonest bacterial organism was *S.pneumoniae* (41%), followed by *H. influenzae* b (6%), and the commonest virus identified was adenovirus (16%), followed by human bocavirus (HBoV) (4%), either as single or co-infection. In total, 3 out of the 4 children with HBoV were aged over 5 years. The infant with HBoV infection was 6 months old, co-infected with CMV and was also HIV-infected. Two of the 3 children with viral co-infections were HIV-infected. Bacterial/viral co-infection occurred in 9 children (9%), of whom 4 (44%) were HIV-infected, and the commonest was *S pneumoniae*/adenovirus (7 cases). Four out of six of the Hib cases were HIV-infected. *P.jirovecii* was identified in 3 infants. All were HIV-infected, less than 6 months of age, co-infected (with Hib, adenovirus and *C. pneumoniae* respectively) and all died. Four out of 6 of the children with Hib pneumonia were fully immunised, one had received only one dose and in one there was no record of immunisation status.

**Table 2:** Aetiology of radiologically confirmed pneumonia.

	No	HIV+ (%)	Blood culture	Lung aspirate culture	Lung aspirate latex	Lung aspirate PCR
**Bacterial aetiology**						
Total	45		8	2	4	36
*S.pneumoniae*	37	68%	6	1	3	31
*S.pneumoniae*/S.*typhimurium*	2	0%	2	1	0	NT
*H.influenzae* type b	6	67%	0	0	1	5
**Viral aetiology**						
Total	24		NT	NT	NT	24
Adenovirus	15	53%	NT	NT	NT	15
Bocavirus	4	50%	NT	NT	NT	4
Cytomegalovirus	3	100%	NT	NT	NT	3
**Atypical**						
*Chlamydia pneumoniae*	2	0%	NT	NT	NT	2
*Mycoplasma pneumoniae*	0		NT	NT	NT	0
**Pneumocystis jirovecii**	3	100%	NT	NT	NT	3

NT: not tested, PcP: pneumocystis pneumonia, Hib: H*aemophilus influenzae b*, CMV: cytomegalovirus.

**Bacteria:** 2 patients had mixed infection with *S.typhimurium* and *S.pneumoniae* ; 1 had *S.typhimurium* from blood culture and *S.pneumoniae* from blood PCR, and adenovirus from lung aspirate PCR, 1 had *S.typhimurium* from blood and lung aspirate culture and *S.pneumoniae* from lung aspirate PCR, 7 had *S.pneumoniae*/adenovirus, 1 had *S.pneumoniae*/Chlamydia, 1 case with *S.pneumoniae* had *M. tuberculosis* cultured from nasopharyngeal aspirate after induced sputum.

**Pneumocystis:** 1 patient had PcP/Hib, 1 patient had PcP/CMV, 1 patient had PcP/adenovirus.

**Viruses:** 1 adenovirus/CMV, 1 adenovirus/Chlamydia, 1 bocavirus/CMV.

Blood culture was positive in only 8 children, 6 with *S.pneumoniae* and 2 with *Salmonella typhimurium*. Lung aspirate culture was positive in 2 children, one with *S.pneumoniae* (blood culture negative) and one with *S. typhimurium* (blood culture positive). In total 14/95 (15%) of children were bacteraemic by blood culture or blood PCR for pneumococcus or Hib. There was no reported prior antibiotic usage in the two cases that were lung aspirate culture positive, but one of them had received the antimalarial sulphadoxine-pyrimethamine prior to admission. Prior antibiotics were reported in 23/36 (64%) with bacteria identified by PCR and in 26/59 (44%) without bacteria identified by PCR (p = 0.1, Chi-squared test). Children with a confirmed bacterial pneumonia (either singly or co-infected), were significantly less likely to have signs of grunting or require oxygen therapy than children without confirmed bacterial pneumonia (Table 3).

**Table 3:** Clinical variables in children with confirmed bacterial pneumonia and children with pneumonia without bacterial confirmation.

Clinical features	Bacterial aetiology	Other aetiology	p value
Increased work of breathing	31/45 (69%)	35/47 (75%)	NS
Grunting	17/46 (37%)	28/49 (57%)	0.05
Crackles	32/45 (71%)	40/48 (83%)	NS
Oxygen therapy	8/45 (18%)	20/46 (44%)	0.008
Temperature>38°C	28/46 (61%)	34/49 (69%)	NS
Oxygen saturation<90%	4/42 (10%)	13/48 (27%)	0.06
Wasting (wt-for-ht Z score<−3)	4/35 (11%)	3/41 (7%)	NS
Stunting (ht-for-age Z score<−3)	11/45 (24%)	9/46 (20%)	NS
Death	2/46 (4%)	4/49 (8%)	NS

NS = not significant.

### Pneumococcal bacterial loads

For children with positive lung aspirate or blood PCR for pneumococcal DNA, pneumococcal bacterial loads were available. Lung aspirate bacterial loads (n = 31) ranged from 2.19×10^2^ to 1.6×10^8^ copies/ml, median 1.25×10^4^, IQR 2.22×10^3^–1.22×10^5^ copies/ml. Blood bacterial loads (n = 11) ranged from 1.22×10^2^ to 1.54×10^6^ copies/ml, median 3.36×10^2^, IQR 1.60×10^2^–1.62×10^3^ copies/ml. Blood and lung aspirate median bacterial loads were higher in non-survivors than in survivors, but these differences were not significant (Blood: 11,047 versus 283 copies/ml and lung aspirate: 247,217 versus 10,869 copies/ml respectively). Lung aspirate bacterial loads were lower in HIV-infected children and blood bacterial loads were higher, but these differences were not significant (Lung aspirate: 8,986 versus 39,859 copies/ml and blood: 831 versus 283 copies/ml respectively). There was no correlation between lung and blood bacterial loads.

### Complications and outcome

Following lung aspiration, two patients (2%) had a pneumothorax on the same side as the aspiration. One presented with worsening respiratory distress about 4 hours after aspiration. A large tension pneumothorax was diagnosed. An intercostal chest drain was inserted and the child did well. The other was a small crescent of air in the pleural space detected on post-aspiration CXR and did not require further management.

Six children died, and all were HIV-infected. Three had PcP identified with co-infections and the other three without PcP were severely malnourished: one aged 1.4 years had a height-for-age Z score of −4.3 and a weight-for-height Z score of −2.4 and developed convulsions during his hospital stay; one aged 6 months had co-infection with HBoV and CMV and had a height-for-age Z score of −3.1; one aged 1.3 years had kwashiorkor, was infected with *S.pneumoniae*, and had a weight-for-height Z score of −2.3.

## Discussion

We have demonstrated that lung aspirate PCR significantly improves the diagnosis of radiological pneumonia from 9/95 (10%) identified by blood and lung aspirate culture to 59/95 (62%) identified by blood and lung aspirate culture and lung aspirate PCR. Co-infection occurs commonly in both HIV-infected and HIV-uninfected children. We provide the first report of HBoV detected from lung aspirate samples in children with radiologically confirmed pneumonia.

The strengths of our study were that we performed PCR to improve diagnosis of both viral and bacterial aetiologies. We performed lung aspirate culture and PCR as well as blood culture; most studies of pneumonia aetiology have previously only used blood culture to identify bacterial aetiologies. Lung aspiration was performed in a select group of children with pneumonia (22%), therefore our findings do not necessarily represent the spectrum of aetiology of radiological pneumonia in this population. Selecting only children with focal, lobar or segmental consolidation meant that most cases of PcP were likely to be excluded, which probably accounts for the lower overall case fatality rate in our study than has been reported in comparable studies [11]. The median age of our group was higher than that of children with pneumonia presenting to Queen Elizabeth Central Hospital [11]; pathogens that commonly occur in infants such as RSV and PcP may therefore be under-represented in our study. Lung aspirate PCR was only designed to identify a small number of bacteria, and did not identify Gram negative organisms such as *Klebsiella* or non-typhoidal salmonella. Validated PCR assays for *Klebsiella* or non-typhoidal salmonella or *Staphylococcus aureus* were not available in our laboratory.

The finding that PCR was much more sensitive than culture in children who had lung aspiration performed is likely to reflect the frequent use of antibiotics prior to presentation as well as the inherent increased sensitivity of the test over culture. The fact that urine was not tested for antibiotic activity may have resulted in a significant under-reporting of previous antibiotic exposure, and may have contributed to the low culture positivity rate despite positive bacterial PCR. An organism identified in a sample obtained directly from consolidated lung is more likely to be the causative agent than are organisms retrieved from samples such as from nasopharyngeal aspirates and even from bronchoalveolar lavage because of the risk of contamination of these samples with carriage isolates [8]. Nasopharyngeal carriage of pneumococcus is very common among well infants in regions such as Malawi [12]. A limitation of RT-PCR compared to culture is that it does not provide antibiotic susceptibility data. Serotype data can now be provided from lung aspirate samples using Luminex technology [13], or PCR [14], [15] but these assays were not available to us at the time of the study.

There are now over 35 reported studies of pneumonia in children where lung aspirate has been used [4], [5], [6], [10]. In the review of the published series up to 1999 the pneumothorax complication rate was 3.3%. There are seven deaths reported from 6001 lung aspirates, a mortality of approximately 1 in 850, although the deaths were not thought to be directly attributable to lung aspiration [4]. Our data are consistent with a study from Finland using lung aspirate for the diagnosis of childhood pneumonia, in which lung aspirate disclosed the aetiology in 59% [6]. That study however had a significantly higher complication rate, with pneumothorax developing in 18% of children, compared with the complication rate in our study of 2%. It must be emphasized that lung aspiration is not recommended in a child suspected to be suffering from PcP, because of the increased risk of pneumothorax [16]. The relatively low number of cases with Hib pneumonia reflects the effect of Hib immunisation in the primary immunisation schedule.

HBoV has been associated with clinical illness in many studies, but it often occurs as a co-infection [17], [18]. This has led to the debate as to whether HBoV is a true pathogen or an innocent bystander. A recent study by Don et al. [19] suggests that the host immune system recognises HBoV as a pathogen by eliciting significant antibody responses. HBoV has also been associated with pneumonia requiring hospitalization in young children in Thailand [20]. Our study is the first study to report HBoV detection in lung aspirate samples in children with radiological pneumonia, strongly suggesting that the infection arose from the lower airways as opposed to merely colonising the nasopharynx. A recent study from Finland found high concentrations of HBoV in induced sputum, also suggesting that the virus may have originated from the lower airways [21].

In a recent study from Kenya, 56% of infants and children who presented with severe pneumonia had one or more respiratory viruses detected on nasal wash samples by PCR [22]. In that study, 4.7%of children were bacteraemic, compared to 15% in our study. In another recent study from Mozambique, 49% of nasopharyngeal aspirate samples from children under 5 years with severe pneumonia had at least one respiratory virus detected by PCR [23]. Viral infection was more prevalent in HIV-infected children, and in-hospital mortality was highest in children with invasive bacterial co-infection and HIV infection [23]. Given that all the children reported in these studies fulfilled the WHO criteria for severe pneumonia, and would therefore have received parenteral antibiotics, there is now an urgent need for reliable, affordable point of care diagnostics to help differentiate bacterial from viral aetiologies in an attempt to promote judicious antibiotic prescribing. We previously reported the performance of five diagnostic biomarkers to predict serious bacterial infection (SBI) in African children [24]. In the current study reported here, median C-reactive protein and procalcitonin levels on admission were significantly higher in children with confirmed bacterial pneumonia than in children with pneumonia without bacterial confirmation, but there was a degree of overlap (data not shown). A larger study assessing the performance of biomarkers of SBI in all children presenting with severe pneumonia is now indicated, augmented by molecular diagnostic testing to enhance diagnosis of bacterial infection. This will allow a more accurate prediction of the utility of biomarkers to guide antibiotic prescribing.

We have shown that lung aspirate PCR significantly increases diagnostic yield in children with focal, segmental or lobar consolidation. In keeping with recent studies from the developing world using PCR for virus detection, our study adds to the growing body of evidence that *S.pneumoniae* and respiratory viruses contribute significantly to the aetiology of childhood pneumonia in Africa. We provide the first report of HBoV from lung aspirate samples suggesting a pathogenic role for this virus in radiologically confirmed pneumonia.

## Methods

### Ethics statement

Ethical approval was obtained from both The College of Medicine Research and Ethics Committee and the Liverpool School of Tropical Medicine Research and Ethics Committee. Information was provided in English and Chichewa. Written consent was obtained from all participants.

### Study patients and procedures

A prospective, observational study of the cytokine profile and genetic determinants of invasive pneumococcal disease (children presenting with pneumonia or meningitis) was undertaken at Queen Elizabeth Central Hospital (QECH), Blantyre, between 1^st^ April 2004 and 30^th^ October 2006. The details of the study participants and recruitment procedures have been previously reported [25], [26], [27]. All children screened for inclusion in the present study had a clinical diagnosis of pneumonia only, and only those with radiologically confirmed pneumonia with focal, segmental or lobar pneumonia were enrolled. Routine immunisation during the period of this study included neonatal BCG, DPT-Hep B and Hib conjugate vaccine at 6, 10 and 14 weeks. Hib vaccine was introduced in Malawi in 2002 and pneumococcal conjugate vaccine was not available throughout the study period.

Clinical data collected on enrolment to the study following written informed consent included prior or current treatment with antibiotics or antimalarials. Chest radiographs were performed on admission and only children with focal, lobar or segmental consolidation were recruited. Arterial oxygen saturation (SpO_2_) when breathing air was measured with pulse oximetry and monitored regularly along with vital signs throughout the hospital stay by nursing staff according to research ward protocols. Urine was not tested for antibiotic activity. All children were reviewed by the study clinicians at least twice per day.

Blood (1–2 ml) was taken for culture prior to commencement of antibiotics and cultured using the BacT/Alert 3D automated system (BioMerieux, France). All isolates were identified using standard diagnostic techniques and antibiotic susceptibility was tested using the Kirby-Bauer disk diffusion method. HIV testing was done following a separate written informed consent with pre- and post-test counselling provided by a trained nurse counsellor, for those who accepted testing. HIV status was determined in all patients. In children ≥18 months, HIV status was assessed using at least two of the following tests; Unigold (Trinity Biotech, Ireland), Serocard (Trinity Biotech, Wicklow, Ireland), or Determine-HIV (Abbott Laboratories, Illinois, USA). In children under 18 months, and those with discordant antibody tests, HIV status was determined using Amplicor HIV-1 DNA (Roche Diagnostics, USA).

### Lung aspiration procedure

Single needle aspiration of consolidated lung was performed prior to commencement of antibiotics in hospital after written informed consent in patients that fitted select criteria: chest radiograph immediately available showing an area of consolidation adjacent to the chest wall, no hyperinflation and *Pneumocystis* pneumonia (PcP) not suspected. Children with severe hypoxia were not excluded. The area of consolidation was confirmed by auscultation and percussion. After aseptic cleaning of the skin, a 23-guage needle attached to a 5 ml syringe containing 2 ml of sterile normal saline was advanced into the area of consolidation. The needle was removed whilst maintaining continuous suction and the sterile saline in the syringe was used to facilitate removal of the lung fluid and debris from the syringe and needle. A chest radiograph was performed routinely after aspiration within one hour, or at any other time when indicated if an air leak was suspected. Paediatric chest drain kits with a range of sizes were always available on the ward.

Lung aspirate fluid was cultured using the BacT/Alert 3D automated system and the remainder of the sample was stored at −80°C until analysis. Bacterial DNA for *H influenzae* and *S pneumoniae* was amplified and quantified using a real-time PCR assay using capsulation (*bexA*), and pneumolysin (*ply*) gene targets specific for, *H. influenzae*, and *S. pneumoniae* respectively using the ABI 7700 Sequence detection system (Taqman), which has been shown to improve non-culture diagnosis and case ascertainment [25],[28]. Real-time quantitative PCR for CMV was performed using the ABI 7500 Sequence detection system (Taqman) using primers which have been previously described [29]. Real-time PCR for *Pneumocystis jirovecii* amplified a section of the large sub-unit of the mitochondrial RNA. Qualitative PCR was also performed on the lung aspirate fluid for Respiratory Syncytial Virus (RSV), Human metapneumovirus (hMPV), Influenza A+B, Parainfuenza virus 1–4, Coronavirus (HKU-1, NL63, OC43), Rhinovirus, Adenovirus, *Chlamydia pneumoniae*, *Mycoplasma pneumonia*e, and Human Bocavirus (HBoV). Briefly, RNA and DNA was extracted using commercial kits (Qiagen, Basingstoke , UK). Extracted RNA was used in a duplex RT-PCR method for RSV nucleocapsid and hMPV matrix gene detection using primers previously described [30], [31]. A multiplex influenza RT-PCR was performed to identify Influenza A and B and a second multiplex RT-PCR was utilized for the detection of parainfluenza virus types 1–4 [32]. Adenovirus, Chlamydia and *M. pneumoniae* were detected using previously published protocols [33]. The HCoV genomes were amplified separately by RT-PCR using previously published primers for; HCoV NL63 [34], HCoV HKU1 [35], HCoV229E, and HCoVOC43 [36]. Bocavirus detection used the primers published previously [37] and rhinovirus detection used the multiplex method described by Choi [36]. The multiplex PCR assays for these respiratory viruses have been used by our group in previous studies [38], [39], [40]. Lung aspirate fluid was not cultured for *Mycobacterium tuberculosis*.

### Definitions

#### *Hypoxia* was defined as SpO_2_ <90%

*Confirmed bacterial pneumonia* was defined as: Radiological evidence of pneumonia (focal, segmental or lobar consolidation) plus one or more of the following: blood or lung aspirate culture positive, lung aspirate positive for pneumococcal polysaccharide antigen or Hib antigen or PCR positive for *S. pneumoniae* or *H. influenzae* b. A cut-off value of >100 copies/ml was used to determine a positive PCR in either blood or lung aspirate. This cut-off value was used based on a previous study using pneumococcal DNA to differentiate between carriage and disease in cases and controls (Carrol ED, unpublished).

*Increased work of breathing:* Recession, grunting, nasal flaring, or use of accessory muscles of respiration.

### Statistical analysis

Median levels of pneumococcal bacterial loads were compared between two groups using the Mann-Whitney U test. Tests for association between binary variables were performed using Fisher's exact test. Reported p values are two-tailed, and the 5% significance level was used to infer statistical significance. Statistical Package for Social Sciences (SPSS), version 15.0 (Illinois, USA) was used for all analyses.
